# Supplementary material for: Porites superfusa mortality and recovery from a bleaching event at Palmyra Atoll, USA
Source: PeerJ. 2017 May 2;5:e3204. doi: 10.7717/peerj.3204 (PMC5417065; doi:10.7717/peerj.3204)
Supplement: Table S1 — Year transitions (2009–2010, 2010–2011, 2011–2012) are recorded because changes in numbers of colonies occurred from one year survey to the next. No resurrected recruits were counted in the 2009 to 2010 year transition because in order to ‘resurrect’ the colony must have at least three years of study. All rows are in number of colonies, except for the mean initial size of colonies in cm2. [file peerj-05-3204-s001.docx]

| Supplementary Table 1. |  |  |  |
| --- | --- | --- | --- |
|  |  |  |  |
| Year Transition | 2009 - 2010 | 2010 - 2011 | 2011 - 2012 |
| Initial size of colonies (cm^2^, Mean + Stdv) | 13.59 + 23.33 | 5.4 + 4.472 | 8.25 + 9.171 |
| Initial colony tally | 721 | 376 | 526 |
| True recruits (Number of colonies) | 71 | 199 | 187 |
| Resurrected recruits (Number of colonies) | n/a | 83 | 18 |
| Growth (Number of colonies) | 73 | 163 | 139 |
| Partial mortality (Number of colonies) | 192 | 52 | 162 |
| Complete mortality (number of colonies) | 416 | 121 | 196 |

**Supplementary Table 1.** Table of demographic changes tallied by year and number of colonies. Year transitions (2009-2010, 2010-2011, 2011-2012) are recorded because changes in numbers of colonies occurred from one year survey to the next. No resurrected recruits were counted in the 2009 to 2010 year transition because in order to ‘resurrect’ the colony must have at least three years of study. All rows are in number of colonies, except for the mean initial size of colonies in cm^2^.
