# Supplementary material for: Porites superfusa mortality and recovery from a bleaching event at Palmyra Atoll, USA
Source: PeerJ. 2017 May 2;5:e3204. doi: 10.7717/peerj.3204 (PMC5417065; doi:10.7717/peerj.3204)
Supplement: Supplemental Information 4 [file peerj-05-3204-s004.rtf]

TukeyHSD(model6, "as.factor(years)", ordered=FALSE)  Tukey multiple comparisons of means    95% family-wise confidence levelFit: aov(formula = size_delta ~ as.factor(years) + site + size_start)$`as.factor(years)`                diff        lwr        upr p adj1011-0910  1.3262044  1.1593278  1.4930810     01112-0910  0.6455031  0.4923524  0.7986538     01112-1011 -0.6807013 -0.8430464 -0.5183562     0Warning message:In replications(paste("~", xx), data = mf) :  non-factors ignored: size_start> TukeyHSD(model6, "site", ordered=FALSE)  Tukey multiple comparisons of means    95% family-wise confidence levelFit: aov(formula = size_delta ~ as.factor(years) + site + size_start)$site          diff          lwr       upr     p adj2-1  0.1699007 -0.007279693 0.3470812 0.06567463-1  0.1000283 -0.133933034 0.3339897 0.68917294-1  0.3486747  0.155559626 0.5417899 0.00002333-2 -0.0698724 -0.310180952 0.1704361 0.87726794-2  0.1787740 -0.021983872 0.3795319 0.10058574-3  0.2486464 -0.003640612 0.5009334 0.0551034Warning message:In replications(paste("~", xx), data = mf) :  non-factors ignored: size_start
